# Cold Chain Logistics Management of Medicine with an Integrated Multi-Criteria Decision-Making Method

**DOI:** 10.3390/ijerph16234843

**Published:** 2019-12-02

**Authors:** Zhi Wen, Huchang Liao, Ruxue Ren, Chunguang Bai, Edmundas Kazimieras Zavadskas, Jurgita Antucheviciene, Abdullah Al-Barakati

**Affiliations:** 1 Business School, Sichuan University, Chengdu 610064, China; wenzhi_456789@163.com (Z.W.); renruxuerrx@163.com (R.R.); 2 Faculty of Computing and Information Technology, King Abdulaziz University, Jeddah 21589, Saudi Arabia; aaalbarakati@kau.edu.sa; 3 Andalusian Research Institute in Data Science and Computational Intelligence (DaSCI), University of Granada, 18071 Granada, Spain; 4 School of Management and Economics, University of Electronic Science and Technology of China, Chengdu 610054, China; cbai@uestc.edu.cn; 5 Institute of Sustainable Construction, Vilnius Gediminas Technical University, LT-10223 Vilnius, Lithuania; edmundas.zavadskas@vgtu.lt; 6 Department of Construction Management and Real Estate, Vilnius Gediminas Technical University, LT-10223 Vilnius, Lithuania; jurgita.antucheviciene@vgtu.lt

**Keywords:** clinical decision-support systems, multiple criteria decision-making, probabilistic linguistic term set, stepwise weight assessment ratio analysis (SWARA), combined compromise solution (CoCoSo), drug cold chain logistics

## Abstract

Medicine is the main means to reduce cancer mortality. However, some medicines face various risks during transportation and storage due to the particularity of medicines, which must be kept at a low temperature to ensure their quality. In this regard, it is of great significance to evaluate and select drug cold chain logistics suppliers from different perspectives to ensure the quality of medicines and reduce the risks of transportation and storage. To solve such a multiple criteria decision-making (MCDM) problem, this paper proposes an integrated model based on the combination of the SWARA (stepwise weight assessment ratio analysis) and CoCoSo (combined compromise solution) methods under the probabilistic linguistic environment. An adjustment coefficient is introduced to the SWARA method to derive criteria weights, and an improved CoCoSo method is proposed to determine the ranking of alternatives. The two methods are extended to the probabilistic linguistic environment to enhance the applicability of the two methods. A case study on the selection of drug cold chain logistics suppliers is presented to demonstrate the applicability of the proposed integrated MCDM model. The advantages of the proposed methods are highlighted through comparative analyses.

## 1. Introduction

Cancer has always been a serious threat to human health. To reduce the incidence of cancer, one important means is to use medicines, such as vaccines, to prevent cancer. However, medicines are special commodities, and their logistics links are quite different from general logistics. Any improper operation in the process of cold chain logistics of medicines may have a significant impact on the quality of medicines and endanger the safety of drug use. This makes the selection of a safe and effective drug cold chain logistics supplier for major pharmaceutical enterprises key to ensuring the safety of refrigerated medicines.

Drug cold chain logistics is a supply chain system that stores and transports medicines from production point to use point at the recommended temperature to ensure the quality of medicines [1]. Different from a general food cold chain logistics system, drug cold chain logistics has many characteristics, such as multi-batch, small batches, timeliness, high operating costs, high coordination of all links in the cold chain, unpredictability, strict qualification examination of business enterprises, high requirements for drug quality standards, and difficult monitoring. Hence, it has requirements, including high standards, high investment, high precision, strict supervision, and high-quality personnel. In recent years, with the innovation of medical technology and new drug research and development technology, the number of cold-chain medicines requiring cryopreservation has increased. However, due to the particularity of the operating environment and equipment requirements, drug cold chain logistics is facing greater risks than other logistics activities. Hence, from the perspective of risk avoidance, through the evaluation of the key risk factors existing in the drug cold-chain logistics suppliers, selecting the drug cold-chain logistics supplier with the lowest comprehensive risk and then realizing the effective control of the risk of drug cold-chain logistics is of great significance to ensure the quality and safety of medicines in enterprises. However, few studies in the literature [1,2] have researched the evaluation and selection of drug cold chain logistics suppliers.

Language is a suitable way to express human cognition and experts can conduct evaluation based on a given linguistic term set (LTS). However, for the evaluation of some complex problems, experts usually hesitate between several linguistic terms because of some vague or uncertain factors. The hesitant fuzzy linguistic term set (HFLTS) [3], as a generalized form of fuzzy linguistic approach, is an effective tool for expressing several linguistic terms at the same time. However, when expressing the evaluation information of most experts with several linguistic terms, the experts may have different preferences for different linguistic terms, which leads to the limitation of the HFLTS in expressing experts’ cognition. In this regard, the probabilistic linguistic term set (PLTS) [4] was developed, which allocates corresponding probabilities to each linguistic term and thus can express complex linguistic information flexibly. Since the evaluation of cold-chain logistics suppliers is qualitative and complex, it is appropriate to use the PLTS as an evaluation tool to express experts’ opinions.

As the selection of drug cold chain logistics suppliers usually involves the evaluation of multiple alternatives under multiple criteria, it is a typical multiple criteria decision-making (MCDM) problem that requires the use of an appropriate MCDM model to solve the problem [5]. At present, different MCDM models have been used to solve the problems of selecting the optimal renewable energy [6], selecting a chief accounting officer [7], evaluating the service quality [8], selecting the best green capacity investment project [9], and evaluating the market segment [10]. Furthermore, various MCDM methods have been extended to solve the decision-making problems in different fields under the probabilistic linguistic environment. For example, Wu and Liao [11] combined the QFD (quality function deployment) and PL-ORESTE (organísation, rangement et Synthèse de données relarionnelles, in French) method and then evaluated the innovative product design based on customer requirements; Wu et al. [12] proposed the PL-MULTIMOORA (multiplicative multiobjective optimization by ratio analysis) method and then applied it to select shared karaoke television brands; Liao et al. [13] developed a procedure of the PL-ELECTRE (ELimination Et Choix Traduisant la REalite, in French) III method to evaluate the nurse–patient relationship; Wu and Liao [14] introduced a comprehensive multiple criteria group decision-making method, which integrated the probabilistic linguistic information with the GLDS (gained and lost dominance score) method to select an optimal green enterprise; Yu et al. [15] developed the PL-PROMETHEE (preference ranking organization method for enrichment evaluation) method for the evaluation of meteorological disaster risk; and Liu and Teng [16] put forward an extended PL-TODIM (an acronym in Portuguese of interactive and multicriteria decision making) method for the selection of products.

In the process of solving MCDM problems, the determination of criteria weights and the ranking of alternatives are two important aspects. The SWARA (stepwise weight assessment ratio analysis) method [17] is an effective method to determine the weights of criteria. Compared with the commonly used AHP (analytic hierarchy process) method, this method does not need a large number of pairwise comparisons and has high consistency. Compared with the BWM (best worst method) [18], this method does not need to solve complex linear objective functions, has less computational complexity, and is easy to understand. To ensure the reliability of the MCDM results, in this study, we introduce an adjustment coefficient to derive the criteria weights. In addition, the CoCoSo (combined compromise solution) method [19] has high stability and reliability regarding the ranking of alternatives. The deletion or addition of alternatives has less impact on the final ranking results obtained by this method than TOPSIS (technique for order preference by similarity to ideal solution), VIKOR (visekriterijumska optimizacija i kompromisno resenje), and other MCDM models. However, the final aggregation operator in this method has shortcomings. In this respect, the DNMA (double normalization-based multiple aggregation) method [20] can effectively overcome the shortcomings of the original CoCoSo method. Hence, in this study, we propose an improved CoCoSo method based on the DNMA method.

To sum up, because the SWARA and CoCoSo methods have their own advantages in determining the weights of criteria and the ranking of alternatives, we extended the SWARA method and improved the CoCoSo method to a probabilistic linguistic environment to form an integrated MCDM model to solve the selection problem of drug cold chain logistics suppliers. This study aimed to:

1. Analyze the defects of the final aggregation operator in the original CoCoSo method and propose a new integration function to improve the CoCoSo method;
2. Introduce an adjustment coefficient to the SWARA method to make the criteria weights reasonable;
3. Develop an integrated MCDM model based on the combination of the SWARA and CoCoSo methods under the probabilistic linguistic environment; and
4. Apply the developed integrated MCDM model to select the optimal drug cold chain logistic suppliers for pharmaceutical manufacturing enterprises in China, and then highlight the advantage of the PL-CoCoSo method by comparative analysis.

This study is organized as follows: Section 2 briefly reviews the literature on drug chain logistics supplier selection, the concepts of PLTSs, and the idea of the SWARA and CoCoSo methods. Section 3 proposes an improvement of the CoCoSo method with a new integration function. Section 4 presents an integrated MCDM model based on the combination of the PL-SWARA and PL-CoCoSo methods. A case study concerning the risk evaluation and selection of drug cold chain logistic suppliers and the relative comparative analysis is given in Section 5. Final conclusions are drawn in Section 6.

## 2. Preliminaries

In this section, we briefly review the literature on drug chain logistics supplier selection, the concepts of PLTSs, and the implementation steps of the SWARA and CoCoSo methods.

### 2.1. Literature Review on Drug Chain Logistics Supplier Selection

Safety and quality assurance in the process of drug transportation is very important for both consumers and pharmaceutical companies. A rational selection of drug logistics suppliers is a hot topic for researchers and practitioners. In this regard, many achievements have been obtained over the past few years. For example, Kulshrestha et al. [21] used the AHP to evaluate the performance of different suppliers of various herbal drugs. The results showed that it is important to include all factors affecting the quality of herbal drugs into performance evaluation. Asamoah et al. [22] used the AHP method to determine the weights of criteria for the selection of suitable suppliers of artemether-lumifen antimalarial drug raw materials for research institutes, and identified the best suppliers of active drug ingredients (API) and the best suppliers of excipients, respectively. The research used the three criteria of quality, price, and reliability/capacity to select the best supplier for a pharmaceutical manufacturing firm in Ghana. Gholamhossein et al. [23] employed an MCDM model to select a suitable supplier for Iranian pharmaceutical companies based on an analysis of distributed questionnaire data. Alinezad et al. [24] combined the QFD and fuzzy AHP method to select a supplier for a pharmaceutical company, in which the QFD method was applied to select suppliers, and the fuzzy AHP method was used to determine the weights of criteria. The study measured the requirements of the organization with some criteria, such as quality, supplier standing, delivery time, and cost. Forghani et al. [25] implemented the PCA (principal component analysis) method to screen out the most important supplier selection criteria. By a TOPSIS-like ordering performance technology, each supplier’s important value for each product was obtained as input value to plan the supplier selection of pharmaceutical companies.

From the above studies, we can see that most studies were about the selection of drug suppliers of pharmaceutical companies, and few literatures used comprehensive decision-making methods to solve the selection of logistics suppliers of pharmaceutical companies. As we know, many medicines are special in nature and require higher storage conditions, so their logistics links are quite different from the requirements of general logistics. The drug cold chain logistics is a supply chain system to ensure the quality of refrigerated drugs. In the existing research of cold chain logistics of medicine, Sinha et al. [1] evaluated the cold chain and logistics management of an immunization program by the GOI monitoring format. The results showed that proper maintenance of the cold chain and management of vaccine logistics can enhance the quality of an immunization program. Chatterjee and Pandey [2] evaluated the drug cold chain from the perspective of risk analysis in which the flow supplier was evaluated and the risk avoidance methods were given. As far as we know, there is no study considering the decision-making problem of cold chain logistics management on medicine. Keeping this in mind, more research about the evaluation and selection of cold chain logistics suppliers should be conducted.

### 2.2. Probabilistic Linguistic Term Set

In 2012, Rodríguez et al. [3] proposed the concept of HFLTS, which is an ordered finite subset of consecutive linguistic terms. It can express the hesitation of experts through several linguistic terms with the same weight. To enhance its applicability, Liao et al. [26] defined the HFLTS in a mathematical form: Let $S=\{s_{\alpha }|\alpha =0,1,\cdots ,2\tau \}$ be a linguistic term set and x∈X be fixed. An HFLTS on X can be represented as $H_{S}=\{<x,h_{S}(x)>|\;x\in X\}$, where the hesitant fuzzy linguistic element (HFLE) $h_{S}(x)=\{s_{\alpha_{l}}(x)|s_{\alpha_{l}}(x)\in S;\;\alpha_{l}\in \{0,1,\cdots ,2\tau \};\;l=1,2,\cdots ,L\}$ is a set of possible linguistic terms of the linguistic variable x to S.

Because experts’ preferences for different linguistic terms are different in most cases, to make the evaluation information expressed by experts more consistent with their cognition, Pang et al. [4] generalized the HFLTS and proposed the PLTS, which adds the corresponding probability to each linguistic term. The PLTS can be expressed as $H_{S}(p)=\{<x,h_{S}(p)>|\;x\in X\}$, where the probabilistic linguistic element (PLE) $h_{S}(p)=\{s_{\alpha_{l}}(p_{l})|s_{\alpha_{l}}\in S;\;\alpha_{l}\in \{0,1,\cdots ,2\tau \};\;p_{l}\geq 0;\;l=1,2,\cdots ,L;\;\sum_{l=1}^{L}p_{l}\leq 1\}$. According to the expectation function of $h_{S}(p)$ proposed by Wu et al. [12], as shown in Equation (1), the PLE can be translated into a crisp number to make the operations flexible:

(1) $$E(h_{S}(p))=\sum_{l=1}^{L}\left(\frac{\alpha_{l}}{2\tau }p_{l}\right)/\sum_{l=1}^{L}p_{l}.$$

### 2.3. Stepwise Weight Assessment Ratio Analysis (SWARA) Method

The SWARA method proposed by Kersuliene et al. [17] can reasonably divide the weights of criteria by synthesizing the knowledge and experience of experts. The operation of the SWARA method is not complicated. See Appendix B for the specific implementation steps of the SWARA method.

Compared with other criterion weight determination methods (such as AHP), the computational complexity of the SWARA method is lower, and it has higher consistency [17]. For these advantages, the SWARA method has been applied in different scenarios to solve practical problems, as shown in Table 1.

### 2.4. Combined Compromise Solution (CoCoSo) Method

The CoCoSo method, as a new MCDM model recently proposed by Yazdani et al. [19], first attains utility values of alternatives from different perspectives through different aggregation operators, and then uses an integration function to integrate utility values of each alternative to obtain a compromise solution. See Appendix A for the specific implementation steps of the CoCoSo method.

Owing to the stable and reliable results obtained by this method, and for the sake of enhancing the practical application of this method, the method was extended to an uncertain environment at present, such as the hesitant fuzzy linguistic CoCoSo method [36] and grey CoCoSo method [37].

## 3. The Improved CoCoSo Method

In this section, two defects of the original CoCoSo method regarding its final integration function are described. To overcome these defects, an improved CoCoSo method with a new integration function is proposed based on the DNMA method to aggregate the three subordinate utility values and subordinate ranks with respect to each alternative determined by the CoCoSo method.

### 3.1. Defects of the Final Integration Function in the Original CoCoSo Method

On the one hand, from the three aggregation strategies shown as Equations (A7)–(A9) in the original CoCoSo method, we can find that the value ranges of the three subordinate compromise performance values are $0<T_{i}^{1}<1$, $T_{i}^{2}\geq 2$, $0<T_{i}^{3}<1$. It is obvious that the effect of the values of $T_{i}^{2}$ on the final results is much greater than that of the values of $T_{i}^{1}$ and $T_{i}^{3}$. That is to say, the dimensions of $T_{i}^{2}$, $T_{i}^{1}$, and $T_{i}^{3}$ are different. However, the final integration function of the original CoCoSo method, shown as Equation (A10), does not normalize the three values or assign different weights to the aggregation values, which leads to the values of $T_{i}^{2}$ having a decisive impact on the final results in most cases, and thus it easy for the final results to have low reliability.

**Example** **1.** *Suppose that the performance values of three alternatives obtained by the three aggregation strategies are as follows:*

$$\begin{array}{l} \begin{array}{ccc} \;T_{i}^{1} & T_{i}^{2} & T_{i}^{3} \end{array}\\ \begin{array}{c} a_{1}\\ a_{2}\\ a_{3} \end{array}\left[\begin{array}{ccc} 0.6 & 2 & 0.7\\ 0.4 & 3 & 0.6\\ 0.3 & 4 & 0.5 \end{array}\right] \end{array}$$

From this matrix, we can get the rankings of the alternatives under three aggregation strategies as: $a_{1}>a_{2}>a_{3}$, $a_{3}>a_{2}>a_{1}$, $a_{1}>a_{2}>a_{3}$. According to the compromise performance values of the alternatives computed by Equation (A10), i.e., $a_{1}=2.044$, $a_{2}=2.23$, and $a_{3}=2.443$, we can deduce the final ranking result as $a_{3}>a_{2}>a_{1}$. However, such a ranking result is clearly inconsistent with the reality since the final ranking result is completely dominated by $T_{i}^{2}$ and the results in terms of $T_{i}^{1}$ and $T_{i}^{3}$ that are contrary to that of $T_{i}^{2}$ are neglected. It shows that the optimal solution is not based on the compromise idea.

On the other hand, the final integration function of the original CoCoSo method only considers the performance values of alternatives generated by three aggregation strategies but ignores the rank of each alternative under different aggregation strategies, which may cause irrational results.

**Example** **2.** *Suppose that the subordinate compromise performance values of three alternatives obtained by the three aggregation strategies are as follows:*

$$\begin{array}{l} \begin{array}{ccc} \;T_{i}^{1} & T_{i}^{2} & T_{i}^{3} \end{array}\\ \begin{array}{c} a_{1}\\ a_{2}\\ a_{3} \end{array}\left[\begin{array}{ccc} 0.4 & 2 & 0.8\\ 0.6 & 2.5 & 0.7\\ 0.2 & 3 & 0.6 \end{array}\right] \end{array}$$

From this matrix, we can get the rankings of the alternatives under three aggregation strategies as: $a_{2}>a_{1}>a_{3}$, $a_{3}>a_{2}>a_{1}$, $a_{1}>a_{2}>a_{3}$. The compromise performance values of the alternatives are calculated as $a_{1}=1.928$, $a_{2}=2.283$, and $a_{3}=1.978$, which implies the final ranking result as $a_{2}>a_{3}>a_{1}$. This result is different from the ranking results obtained by the three strategies. Moreover, without considering the subordinate rankings, the values of $T_{i}^{2}$ have a greater impact than those of $T_{i}^{1}$ and $T_{i}^{3}$ on the final ranking result, which leads to the result being unstable and unreasonable.

### 3.2. A New Integration Function for the CoCoSo Method

The double normalization-based multiple aggregation (DNMA) method, as a novel MCDM method, was proposed by Liao and Wu [20]. The final integration function of the DNMA method comprehensively considers the subordinate utility values and the ranks of alternatives, and thus the final ranking result has high reliability. Inspired by this method, we introduce a new function to integrate the three subordinate performance values under three aggregation strategies as follows:

(2) $$T_{i}^{*}=\sum_{v=1}^{3}\sqrt{0.5({\left((T_{i}^{v*})/\underset{i}{\max}T_{i}^{v*}\right)}^{2}+{\left((m-r_{i}^{v})/m\right)}^{2})},\;\mathrm{for}\;i=1,2,\cdots ,m,$$

where $T_{i}^{v*}$ refers to the normalized values of $T_{i}^{v}$ corresponding to alternative $a_{i}^{}$ by vector normalization, and $r_{i}^{v}$ refers to the rank of alternative $a_{i}^{}$ with respect to values of $T_{i}^{v*}$. v refers to the number of aggregation strategies and v=1,2,3.

**Remark** **1.** *Since this function unifies the dimensions of three aggregation strategies in the calculation process, it is no longer necessary to normalize the three aggregation strategy values before using the function.*

**Example** **3.** *For Example 1, we can calculate the compromise performance values of the three alternatives by Equation (2). Then, we have* $a_{1}=2.053$ *,* $a_{2}=1.758$ *, and* $a_{3}=1.708$ *, and thus the final ranking result* $a_{1}>a_{2}>a_{3}$ *can be obtained. This ranking result reduces the decisive influence of the value of $T_{i}^{2}$ on the final result in this example, and the ranking results based on the values of $T_{i}^{1}$ and $T_{i}^{3}$ are fully considered. In this sense, the ranking result deduced by Equation (2) is more in line with the idea of compromise.*

**Example** **4.** *For Example 2, the compromise performance values of the three alternatives, i.e., $a_{1}=1.848$, $a_{2}=2.147$, and $a_{3}=1.616$, can be calculated by Equation (2), and thus the final ranking result is $a_{2}>a_{1}>a_{3}$. The ranking results of alternative $a_{1}$ under the three aggregation strategies are 2, 3, and 1, respectively, and those of alternative $a_{3}$ under the three aggregation strategies are 3, 1, and 3, respectively. In the case of reducing the impact of the value of $T_{i}^{2}$ on the final ranking result, alternative $a_{1}$ should rank higher than alternative $a_{3}$. Hence, the new integration function that considers the compromise performance values and ranks of alternatives is more reasonable than that of the original CoCoSo method.*

## 4. An Integrated MCDM Model Based on the PL-SWARA and PL-CoCoSo Methods

In this section, we extend the SWARA method to the probabilistic linguistic context and employ the PL-SWARA method to derive criteria weights. Then, we extend the CoCoSo method to the probabilistic linguistic context and apply the PL-CoCoSo method to rank alternatives. Afterwards, the procedure of the integrated MCDM model is presented.

### 4.1. Determine the Weights of Criteria Based on the PL-SWARA Method

First, after a series of evaluation criteria being established, an expert or multiple experts ($D_{1},D_{2},\cdots ,D_{k},\cdots ,D_{e}$) will be invited to rank the criteria based on their importance. In this regard, if more than one expert is invited, the ranking results they provide need to be consistent to reduce the deviation of the result.

The importance ranking of the criteria can be determined as $c_{\left(1\right)},c_{\left(2\right)},\cdots ,c_{\left(j\right)},\cdots ,c_{\left(n\right)}$, where $c_{\left(1\right)}$ is the most important criterion in these criteria and $c_{\left(n\right)}$ is the least important one. Next, the probabilistic linguistic evaluation about the importance of criterion $c_{j}(j>1)$ relative to criterion $c_{j-1}$ is provided by an expert or multiple experts according the linguistic term set $S_{1}$:$\{I_{0}:extremely$$unimportant,\;I_{1}:very\;unimportant,\;I_{2}:unimportant,\;I_{3}:moderately\;unimportant,\;I_{4}:slightly\;unimportant\}$. Converting the expert’s probabilistic linguistic evaluation information into PLEs, the expectation values of these PLEs are calculated and normalized by vector normalization to obtain the relative importance of criterion $c_{j}$, $z_{j}^{*}$. In the case of multiple experts, it is necessary to aggregate the values given by different experts by the weighted average aggregation operator shown as Equation (3) and normalized the value by vector normalization to obtain $z_{j}^{*}$:

(3) $$WA\;(E(h_{S}^{j(D_{1})}(p)),E(h_{S}^{j(D_{2})}(p)),\;\cdots ,E(h_{S}^{j(D_{e})}(p)))=\sum_{k=1}^{e}\lambda_{k}E(h_{S}^{j(D_{k})}(p)),\;\mathrm{for}\;j=2,3,\cdots ,n,$$

where $E(h_{S}^{j(D_{k})}(p))$ represents the expectation values of the PLEs under criterion $c_{j}$ corresponding to expert $D_{k}$, and $\lambda_{k}$(k=1,2,⋯,e) represent the weights of experts with $\sum_{k=1}^{e}\lambda_{k}=1$.

To reduce the impact of the uncertainty of criterion weights on the final results, we can introduce an adjustment coefficient of the weight of each criterion according to the expectation value of the PLE for the criterion, and the larger the sum of the difference values between these PLEs is, the smaller the weight of the criterion should be. Hence, by calculating the difference between the expectation values, the adjustment coefficient can be deduced below:

(4) $$AC_{j}=\frac{\sum_{k=1}^{e}\sum_{t=1}^{e}E(h_{S}^{j(D_{k})}(p))-E(h_{S}^{t(D_{k})}(p))}{\sum_{j=2}^{n}\sum_{k=1}^{e}\sum_{t=1}^{e}E(h_{S}^{j(D_{k})}(p))-E(h_{S}^{t(D_{k})}(p))},\;\mathrm{for}\;j=2,3,\cdots ,n,$$

when j=1, $AC_{\left(j\right)}=1$. Because the comparative evaluation between criteria starts from the second criterion, the difference value with respect to criterion $c_{1}$ equals 0.

Afterwards, the subordinate weights of criteria can be derived by the following equation:

(5) $$w_{j}^{\prime }=\{\begin{array}{ll} 1, & j=1\\ \frac{z_{j-1}^{*}+1}{z_{j}^{*}+1}, & j>1 \end{array}.$$

Finally, we can derive the final weight of each criterion by:

(6) $$w_{j}=\frac{AC_{j}w_{j}^{\prime }}{\sum_{j=1}^{n}AC_{j}w_{j}^{\prime }},\;\mathrm{for}\;j=1,2,\cdots ,n.$$

### 4.2. Rank the Alternatives by the PL-CoCoSo Method

The expert or multiple experts are required to evaluate a series of alternatives, $a_{1},a_{2},\cdots ,a_{i},\cdots ,a_{m}$, over a set of criteria, $c_{1},c_{2},\cdots ,c_{j},\cdots ,c_{n}$, based on a linguistic term set provided in advance. If only one expert gives linguistic evaluation information, he/she needs to assign probabilities to each linguistic term to form a probabilistic linguistic decision matrix. If more than one expert gives linguistic evaluation information, the probabilistic linguistic decision matrix can be obtained by aggregating the number of each linguistic term in each expert’s linguistic decision matrix. Then, a probabilistic linguistic decision matrix is obtained as:

$$\left[\begin{array}{cccccc} h_{S}^{11}(p) & h_{S}^{12}(p) & \cdots & h_{S}^{1j}(p) & \cdots & h_{S}^{1n}(p)\\ h_{S}^{21}(p) & h_{S}^{22}(p) & \cdots & h_{S}^{2j}(p) & \cdots & h_{S}^{2n}(p)\\ \vdots & \vdots & \ddots & \vdots & \ddots & \vdots \\ h_{S}^{i1}(p) & h_{S}^{i2}(p) & \cdots & h_{S}^{ij}(p) & \cdots & h_{S}^{in}(p)\\ \vdots & \vdots & \ddots & \vdots & \ddots & \vdots \\ h_{S}^{m1}(p) & h_{S}^{m2}(p) & \cdots & h_{S}^{mj}(p) & \cdots & h_{S}^{mn}(p) \end{array}\right],$$

where $h_{S}^{ij}(p)$ represents the PLE of alternative $a_{i}$ under criterion $c_{j}$.

Next, based on the expectation function given as Equation (1), the expectation value of each PLE in the probabilistic linguistic decision matrix is calculated. Then, we normalize the expectation value under each criterion according to the type of criterion.

For benefit criteria, we have:

(7) $$\hat{E}(h_{S}^{ij}(p))=\frac{\left(E(h_{S}^{ij}(p))-\underset{i}{\min}E(h_{S}^{ij}(p))\right)}{\left(\underset{i}{\max}E(h_{S}^{ij}(p))-\underset{i}{\min}E(h_{S}^{ij}(p)\right)}.$$

For cost criteria, we have:

(8) $$\hat{E}(h_{S}^{ij}(p))=\frac{\left(\underset{i}{\max}E(h_{S}^{ij}(p))-E(h_{S}^{ij}(p))\right)}{\left(\underset{i}{\max}E(h_{S}^{ij}(p))-\underset{i}{\min}E(h_{S}^{ij}(p)\right)}.$$

Based on the weights of criteria derived by the PL-SWARA method, we respectively compute the arithmetically weighted sum, $AW_{i}$, and the geometrically weighted sum, $GW_{i}$, for each alternative by the following equations:

(9) $$AW_{i}=\sum_{j=1}^{n}(w_{j}\hat{E}(h_{S}^{ij}(p))),$$

(10) $$GW_{i}=\sum_{j=1}^{n}{\left(\hat{E}(h_{S}^{ij}(p))\right)}^{w_{j}}.$$

Afterwards, through three aggregation strategies, we can combine $AW_{i}$ and $GW_{i}$ and obtain three subordinate compromise performance values for each alternative. The first aggregation strategy stands on the mean of $AW_{i}$ and $GW_{i}$, as shown in Equation (11). The second aggregation strategy stands on the sum of the comparison of $AW_{i}$ and $GW_{i}$ with the worst one, as shown in Equation (12). The third aggregation strategy stands on the balanced compromise of $AW_{i}$ and $GW_{i}$, as shown in Equation (13), and the parameter $\delta^{*}$ is a balance parameter determined by experts according to their preferences. If the experts pay more attention to the comprehensive performances of alternatives, they can assign a larger value to $\delta^{*}$; if the experts pay more attention to the outstanding performances of alternatives, they can give $\delta^{*}$ a smaller value:

(11) $$T_{i}^{1*}=\frac{AW_{i}+GW_{i}}{\sum_{i=1}^{m}\left(AW_{i}+GW_{i}\right)},$$

(12) $$T_{i}^{2*}=\frac{AW_{i}}{\underset{i}{\min}AW_{i}}+\frac{GW_{i}}{\underset{i}{\min}GW_{i}},$$

(13) $$T_{i}^{3*}=\frac{\delta^{*}(AW_{i})+(1-\delta^{*})(GW_{i})}{\delta^{*}\underset{i}{\max}AW_{i}+(1-\delta^{*})\underset{i}{\max}GW_{i}}.$$

Eventually, we utilize the final integration function shown as Equation (2) to attain the final compromise values of alternatives, and determine the optimal alternative in descending order of the final compromise values.

### 4.3. Procedure of the Integrated MCDM Method with the Combination of the PL-SWARA and PL-CoCoSo Methods

Based on the analyses in Section 3.1 and Section 3.2, we can develop an integrated MCDM method which includes the following six steps:

**Step 1.** Determine an MCDM problem that involves multiple alternatives and criteria. An expert or expert group is invited to rank the criteria via pairwise comparisons to obtain the probabilistic linguistic preference information on the importance of criteria. For the expert group, it is necessary to aggregate the information of each expert by Equation (3).

**Step 2.** Calculate the adjustment coefficients by Equation (4) and deduce the subordinate criteria weights by Equation (5).

**Step 3.** Derive the final weights of criteria by Equation (6).

**Step 4.** Ask the expert or expert group to evaluate the alternatives over the criteria according to the given linguistic term set. For the expert group, it is necessary to aggregate the linguistic evaluation information of each expert to form a probabilistic linguistic decision matrix.

**Step 5.** Normalize the decision matrix by Equations (7) and (8). Then, combine those normalization values and the criteria weights derived in Step 3 to compute the arithmetically and geometrically weighted sum by Equations (9) and (10). Next, three aggregation strategies shown as Equations (11)–(13) are used to obtain the subordinate performance values and subordinate rankings of the alternatives.

**Step 6.** Compute the final compromise performance value of each alternative by the integration function given as Equation (2) to attain the final ranking of the alternatives and determine the optimal alternative.

**Figure 1:**
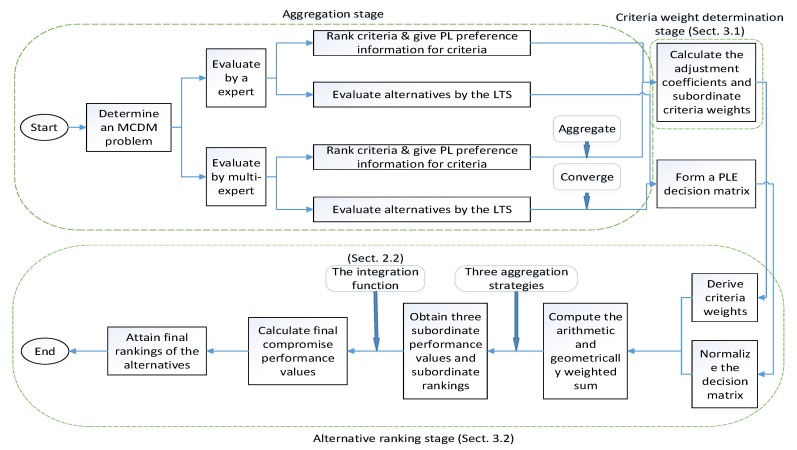
Flowchart of the integrated MCDM model.

The flowchart of this integrated MCDM model is shown in Figure 1.

## 5. Case Study: Risk Evaluation and Selection of Drug Cold Chain Logistics Suppliers

In this section, a case study concerning the selection of cold chain logistics suppliers of medicine is given to demonstrate the applicability of the proposed integrated MCDM model. Some comparative analyses are further provided to validate the advantages of the proposed MCDM method.

### 5.1. Case Description

According to the latest national cancer statistics published by China National Cancer Center in January 2019 (the data of China Cancer Registration Center generally lags behind three years), in 2015, malignant tumors occurred in about 39.29 million people and died in about 2.338 million people, with an average of more than 10,000 people diagnosed with cancer every day (http://www.360doc2.net/wxarticlenew/812065860.html). Thus, it can be seen that cancer has become one of the major public health problems that seriously threaten the health of Chinese people. Vaccines, as an effective drug to reduce the incidence of cancer, have high transportation and storage environment requirements in the processes from production to consumption. The quality is very likely to change if these requirements cannot be met, thus affecting the effect of vaccines, and in serious cases may also endanger the health of vaccine seeders. Therefore, for most drug manufacturers that produce vaccines and other medicines requiring cryopreservation, it is essential to select a drug cold chain logistics supplier with the lowest risk.

R company is a high-tech company in China, with modern pharmaceutical industry as the core, focusing on the integration of biopharmaceutical, dynamic pharmaceutical, manufacturing construction and bioengineering technology application research, development, production, and operation. The company’s monoclonal antibody injection for liver cancer, pseudorabies gene deletion vaccine, and other technical projects have been included in the national key technological innovation projects. The Licartin produced by the company is a monoclonal antibody radioimmunoassay targeting medicine for the treatment of primary liver cancer. As an isotope-labeling medicine, Licartin is radioactive and requires high hardware and software conditions for production, labeling, transportation, distribution, and clinical use. To ensure the quality of its pharmaceutical products, reduce the high cost of medicines during storage and transportation, and improve its competitiveness, R company needs to select a suitable drug cold chain logistics service supplier. Suppose that four experts ($D_{1}$, $D_{2}$, $D_{3}$, $D_{4}$) with the same weight were invited to promote the solution of this MCDM problem. After some screening, six suppliers ($A_{1}$, $A_{2}$, $A_{3}$, $A_{4}$, $A_{5}$, $A_{6}$) were determined in the candidate list. These suppliers are well-known cold chain logistics enterprises in China, and there is not much gap in honor, quality, and service. Considering the high risk of drugs in cold chain logistics, eight key risk factors regarding storage and transportation are identified by the experts as risk evaluation criteria ($c_{1}$, $c_{2}$, $c_{3}$, $c_{4}$, $c_{5}$, $c_{6}$, $c_{7}$, $c_{8}$), as shown in Table 2. The linguistic evaluation information of the alternatives over the criteria was given by the four experts according to the linguistic term set, $S_{2}$:$\{s_{0}:extremely\;low,\;s_{1}:very\;low,s_{2}:\;low,\;s_{3}:slightly\;low,\;s_{4}:medium,\;s_{5}:\;slightly\;high,\;s_{6}:high,$ $s_{7}$: *very high*, $s_{8}:extremely\;high\}$ as shown in Table A1, Table A2, Table A3 and Table A4 in Appendix C.

### 5.2. Using the Integrated MCDM Method to Solve the Case

The specific steps of applying the proposed method to solve the multi-expert MCDM problem of selecting the optimal drug cold chain logistics supplier are as follows:

**Step 1.** A series of alternatives, criteria, and experts involved in the multi-expert MCDM problem were identified in the case description. The experts rank the criteria and get a unified ranking of the importance of the criteria. The ranking is $c_{2}$, $c_{7}$, $c_{1}$, $c_{4}$, $c_{5}$, $c_{3}$, $c_{6}$, $c_{8}$. Then, according to the linguistic term set, $S_{1}$, the pairwise comparisons are made between the latter criterion and the former criterion, and the individual preference information of each expert is displayed in Table A5 in Appendix C.

**Step 2.** We account for the adjustment coefficients for the criteria by Equation (4). Then, the expectation value of each PLE can be calculated based on the expectation function given as Equation (1). After normalizing the expectation values by vector normalization, the relative importance of the criteria $z_{j}^{*}\;(j=2,3,\cdots ,n)$ can be obtained by Equation (3). According to the values of $z_{j}^{*}$, we can deduce the subordinate weights of the criteria by Equation (5).

**Step 3.** Combining the adjustment coefficients, $AC_{j}$, and subordinate weights, $w_{j}^{\prime }$, the final weights of the criteria can be derived by Equation (6). The results are shown in Table 3.

**Step 4.** We aggregate the linguistic evaluation information of the alternatives over the criteria provided by experts into a probabilistic linguistic decision matrix according to the number of linguistic elements used by the experts:

$$\begin{array}{l} {}[\begin{array}{cccc} \left\{s_{3}(0.167),s_{4}(0.5),s_{5}(0.333)\right\} & \left\{s_{5}(0.222),s_{6}(0.445),s_{7}(0.333)\right\} & \left\{s_{2}(0.5),s_{3}(0.5)\right\} & \left\{s_{2}(0.333),s_{3}(0.445),s_{4}(0.222)\right\}\\ \left\{s_{4}(0.375),s_{5}(0.375),s_{6}(0.25)\right\} & \left\{s_{5}(0.8),s_{6}(0.2)\right\} & \left\{s_{1}(0.222),s_{2}(0.333),s_{3}(0.445)\right\} & \left\{s_{3}(0.286),s_{4}(0.428),s_{5}(0.286)\right\}\\ \left\{s_{6}(0.429),s_{7}(0.429),s_{8}(0.142)\right\} & \left\{s_{4}(0.142),s_{5}(0.429),s_{6}(0.429)\right\} & \left\{s_{3}(0.333),s_{4}(0.5),s_{5}(0.167)\right\} & \left\{s_{1}(0.167),s_{2}(0.5),s_{3}(0.333)\right\}\\ \left\{s_{3}(0.25),s_{4}(0.375),s_{5}(0.375)\right\} & \left\{s_{6}(0.286),s_{7}(0.428),s_{8}(0.286)\right\} & \left\{s_{3}(0.444),s_{4}(0.444),s_{5}(0.112)\right\} & \left\{s_{3}(0.333),s_{4}(0.667)\right\}\\ \left\{s_{5}(0.5),s_{6}(0.5)\right\} & \left\{s_{6}(0.222),s_{7}(0.445),s_{8}(0.333)\right\} & \left\{s_{4}(0.571),s_{5}(0.429)\right\} & \left\{s_{2}(0.2),s_{3}(0.4),s_{4}(0.4)\right\}\\ \left\{s_{4}(0.125),s_{5}(0.5),s_{6}(0.375)\right\} & \left\{s_{6}(0.429),s_{7}(0.429),s_{8}(0.142)\right\} & \left\{s_{2}(0.5),s_{3}(0.5)\right\} & \left\{s_{2}(0.167),s_{3}(0.5),s_{4}(0.333)\right\} \end{array}\\ \begin{array}{cccc} \left\{s_{4}(0.5),s_{5}(0.375),s_{6}(0.125)\right\} & \left\{s_{2}(0.667),s_{3}(0.333)\right\} & \left\{s_{3}(0.25),s_{4}(0.5),s_{5}(0.25)\right\} & \left\{s_{0}(0.5),s_{1}(0.5)\right\}\\ \left\{s_{2}(0.286),s_{3}(0.571),s_{4}(0.143)\right\} & \left\{s_{1}(0.3),s_{2}(0.4),s_{3}(0.3)\right\} & \left\{s_{4}(0.125),s_{5}(0.5),s_{6}(0.375)\right\} & \left\{s_{1}(0.25),s_{2}(0.5),s_{3}(0.25)\right\}\\ \left\{s_{1}(0.3),s_{2}(0.3),s_{3}(0.4)\right\} & \left\{s_{3}(0.4),s_{4}(0.6)\right\} & \left\{s_{5}(0.3),s_{6}(0.4),s_{7}(0.3)\right\} & \left\{s_{2}(0.375),s_{3}(0.5),s_{4}(0.125)\right\}\\ \left\{s_{1}(0.286),s_{2}(0.571),s_{3}(0.143)\right\} & \left\{s_{3}(0.375),s_{4}(0.375),s_{5}(0.25)\right\} & \left\{s_{6}(0.5),s_{7}(0.375),s_{8}(0.125)\right\} & \left\{s_{0}(0.4),s_{1}(0.4),s_{2}(0.2)\right\}\\ \left\{s_{3}(0.4),s_{4}(0.2),s_{5}(0.4)\right\} & \left\{s_{4}(0.167),s_{5}(0.333),s_{6}(0.5)\right\} & \left\{s_{3}(0.125),s_{4}(0.375),s_{5}(0.5)\right\} & \left\{s_{1}(0.142),s_{2}(0.429),s_{3}(0.429)\right\}\\ \left\{s_{3}(0.375),s_{4}(0.5),s_{5}(0.125)\right\} & \left\{s_{1}(0.375),s_{2}(0.375),s_{3}(0.25)\right\} & \left\{s_{5}(0.375),s_{6}(0.357),s_{7}(0.25)\right\} & \left\{s_{1}(0.4),s_{2}(0.4),s_{3}(0.2)\right\} \end{array}] \end{array}$$

Computing the expectation value of each PLE in the decision matrix by Equation (1), we can obtain:

$$\left[\begin{array}{cccccccc} 0.521 & 0.764 & 0.313 & 0.361 & 0.578 & 0.292 & 0.5 & 0.188\\ 0.609 & 0.65 & 0.278 & 0.5 & 0.357 & 0.25 & 0.656 & 0.219\\ 0.839 & 0.661 & 0.479 & 0.271 & 0.263 & 0.45 & 0.75 & 0.344\\ 0.516 & 0.875 & 0.459 & 0.458 & 0.232 & 0.484 & 0.828 & 0.1\\ 0.688 & 0.889 & 0.554 & 0.4 & 0.5 & 0.667 & 0.547 & 0.286\\ 0.656 & 0.839 & 0.313 & 0.396 & 0.469 & 0.234 & 0.734 & 0.225 \end{array}\right]$$

According to Equation (8), we can attain the normalization decision matrix as:

$$\left[\begin{array}{cccccccc} 0.985 & 0.523 & 0.873 & 0.607 & 0 & 0.866 & 1 & 1\\ 0.712 & 1 & 1 & 0 & 0.639 & 0.963 & 0.524 & 0.335\\ 0 & 0.954 & 0.272 & 1 & 0.91 & 0.501 & 0.238 & 0\\ 1 & 0.059 & 0.344 & 0.183 & 1 & 0.423 & 0 & 0.868\\ 0.467 & 0 & 0 & 0.437 & 0.225 & 0 & 0.857 & 0.206\\ 0.567 & 0.209 & 0.873 & 0.454 & 0.315 & 1 & 0.287 & 0.423 \end{array}\right]$$

**Step 5.** Based on the weights of the criteria derived in Step 3 and Equations (9) and (10), we can obtain the arithmetically weighted sum, $AW_{i}$, and the geometrically weighted sum, $GW_{i}$. Then, the subordinate performance values and subordinate rankings of the alternatives obtained through the three aggregation strategies are shown in Table 4. Since the value of $GW_{i}$ is obviously larger than that of $AW_{i}$, we suppose that the balance parameter is $\delta^{*}=0.8$ to achieve a better balance effect.

**Step 6.** The integration function given as Equation (2) is utilized to attain the final compromise performance values of the alternatives. The results are displayed in Table 4, and it can be determined that the optimal drug cold chain logistics supplier is $A_{2}$.

### 5.3. Applying other Methods to Solve the Case

#### 5.3.1. Applying the Original CoCoSo Method to Solve the Case

Based on three subordinate performance values of the alternatives calculated by the PL-CoCoSo method, we used the integration function in the original CoCoSo method shown as Equation (A10) to calculate the final compromise performance values of the alternatives. The results are 2.178, 2.177, 1.849, 1.581, 1.326, and 1.807, which implies that the final ranking results of the alternatives are 1, 2, 3, 5, 6, and 4. We can find that this final ranking result is the same as the ranking result under the aggregation strategy, $T_{i}^{2*}$, which indicates that the aggregation strategy, $T_{i}^{2*}$, has a much greater impact on the final results than the aggregation strategies, $T_{i}^{1*}$ and $T_{i}^{3*}$. This can easily lead to unreasonable final results and make the method of finding the compromise solution incapable of being reflected.

#### 5.3.2. Applying the PL-MULTIMOORA Method to Solve the Case

Since both the MULTIMOORA and CoCoSo methods are MCDM models, which integrate three aggregation operators from different perspectives to obtain compromise solutions, this paper applies the PL-MULTIMOORA method proposed by Wu et al. [12] to solve the problem and compares it with the PL-CoCoSo method.

Based on the expectation value of each PLE in the aggregated probabilistic linguistic decision matrix in Section 5.2, a vector-normalized decision matrix is obtained as:

$$\left[\begin{array}{cccccccc} 0.328 & 0.397 & 0.31 & 0.365 & 0.563 & 0.28 & 0.301 & 0.11\\ 0.384 & 0.338 & 0.275 & 0.505 & 0.348 & 0.24 & 0.394 & 0.437\\ 0.529 & 0.343 & 0.474 & 0.274 & 0.256 & 0.432 & 0.451 & 0.601\\ 0.325 & 0.455 & 0.454 & 0.463 & 0.226 & 0.465 & 0.498 & 0.175\\ 0.434 & 0.462 & 0.548 & 0.404 & 0.487 & 0.641 & 0.329 & 0.5\\ 0.414 & 0.436 & 0.31 & 0.4 & 0.457 & 0.225 & 0.441 & 0.393 \end{array}\right].$$

Since the criteria are all cost-type, the third operator in the PL-MULTIMOORA method is not applicable in this case. The subordinate performance values of the alternatives can be computed by the following two aggregation operators:

$$M_{1}(A_{i})=-\sum_{j=1}^{n}w_{j}E^{N}(h_{S}^{ij}(p)),$$

$$M_{2}(A_{i})=\underset{j}{\max}\left\{w_{j}(E^{N}(h_{S}^{ij}(p)))-\underset{i}{\min}E^{N}(h_{S}^{ij}(p))))\right\},$$

where $E^{N}(h_{S}^{ij}(p))$ represents the normalized expectation value of each PLE. The calculation results of $M_{1}(A_{i})$ and $M_{2}(A_{i})$ are (−0.358, −0.373, −0.397, −0.42, −0.437, −0.417) and (0.024, 0.026, 0.032, 0.041, 0.044, 0.034), respectively. The former is ranked in descending order while the latter is ranked in ascending order to get the corresponding ranking results of $r_{1}(A_{i})$ = (1, 2, 3, 5, 6, 4) and $r_{2}(A_{i})$ = (1, 2, 3, 5, 6, 4).

Then, we calculate the final compromise performance values of the alternatives by the integration function:

$$M(A_{i})=M_{1}(A_{i})*\frac{m-r_{1}(A_{i})+1}{m(m+1)/2}-M_{2}(A_{i})*\frac{r_{2}(A_{i})}{m(m+1)/2}.$$

The calculation results are (−0.118, −0.12, −0.131, −0.157, −0.171, −0.138), which indicates that the final ranking results of the alternatives are (1, 2, 3, 5, 6, 4).

**Figure 2:**
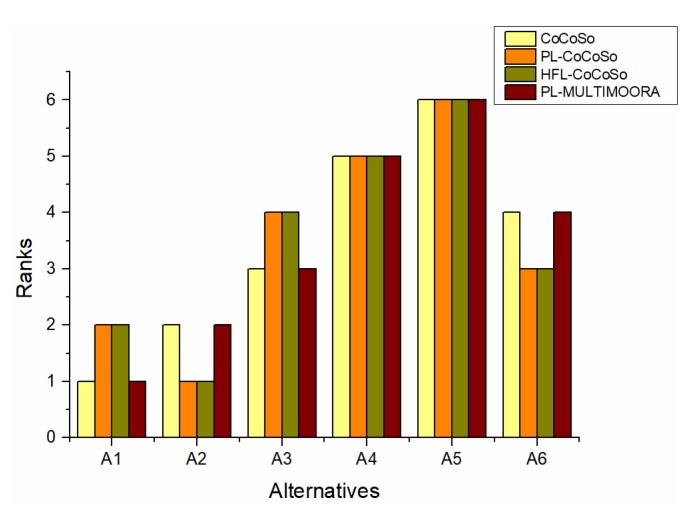
Comparisons between the proposed method and other methods.

According to Figure 2, it is obvious that the results obtained by the PL-MULTIMOORA method are different from those obtained by the PL-CoCoSo method. This may be because of the fact that the MCDM problem in the case only involves the criteria of cost type but does not include the criteria of the benefit type. The PL-MULTIMOORA method is suitable for solving the MCDM problems that contain both cost and benefit types of criteria. By contrast, the application of the PL-CoCoSo method is more extensive.

#### 5.3.3. Applying the HFL-CoCoSo Method to Solve the Case

Since a series of hesitant fuzzy linguistic terms are included in the evaluation of the alternatives under the criteria provided by the experts in the case, we apply the HFL-CoCoSo method proposed by Wen et al. [36], which extends the CoCoSo method to the hesitant fuzzy linguistic environment to solve the case and compare it with the proposed PL-CoCoSo method.

Based on the linguistic evaluation information of the alternatives on the criteria given by the experts in the case description, we convert the linguistic information into HFLEs and the score of each HFLE is calculated through the score function:

$$G(h_{S}(x))=(1-\frac{L(h_{S}(x))\ln(L(h_{S}(x)))}{\left(2\tau +1)\ln(2\tau +1\right)})\times (\frac{1}{L}\sum_{l=1}^{L}\frac{\varphi_{l}}{2\tau }).$$

Then, we aggregate the individual evaluation information of the experts by the weighted average aggregation operator to form the following decision matrix:

$$\left[\begin{array}{cccccccc} 0.514 & 0.689 & 0.323 & 0.325 & 0.517 & 0.271 & 0.468 & 0.059\\ 0.546 & 0.629 & 0.271 & 0.458 & 0.37 & 0.221 & 0.607 & 0.25\\ 0.778 & 0.639 & 0.452 & 0.273 & 0.25 & 0.446 & 0.662 & 0.32\\ 0.493 & 0.807 & 0.41 & 0.477 & 0.223 & 0.431 & 0.748 & 0.082\\ 0.688 & 0.801 & 0.518 & 0.36 & 0.508 & 0.666 & 0.523 & 0.283\\ 0.607 & 0.778 & 0.302 & 0.391 & 0.433 & 0.2 & 0.662 & 0.192 \end{array}\right].$$

Then, we calculate the values of $T_{i}^{v}\;(v=1,\;2,\;3)$ according to the same calculation method as Equations (8)–(13). We can obtain the results, as shown in Table 5.

Suppose that the order of the importance of the three aggregation strategies is $r(T_{i}^{3^{\prime }})≻r(T_{i}^{1^{\prime }})=r(T_{i}^{2^{\prime }})$. Then, the global preference score of alternative $A_{i}$ regarding the aggregation strategy $T_{i}^{v^{\prime }}$, $PS_{v}(A_{i})$, can be calculated by:

$$PS_{v}(A_{i})=\sqrt{0.5\times (0.5\times ({\left(r_{v}(A_{i})\right)}^{2}+{\left(r(T_{i}^{v^{\prime }})\right)}^{2})+\max\{{\left(r_{v}(A_{i})\right)}^{2},{\left(r(T_{i}^{v^{\prime }})\right)}^{2}\})}.$$

According to the three global preference scores of each alternative, three ranks $R_{v}(A_{i})\;(v=1,\;2,\;3)$ are obtained respectively, and the final ranking is obtained according to the sum of the three rankings, $R(A_{i})$, in descending order, as shown in Table 6.

From Figure 2, it can be seen that the ranking result derived by the HFL-CoCoSo method is the same as that derived by the PL-CoCoSo method. On the one hand, it reflects the reasonability of the results obtained by this method. On the other hand, although the results are the same, the calculation process of the HFL-CoCoSo method is more complicated than that of the PL-CoCoSo method. In addition, although the HFL-CoCoSo method considers the relative importance of three aggregation strategies, the final ranking result only summarizes the results of the subordinate ranks, without considering the utility values and ranks at the same time. By contrast, the PL-CoCoSo method is more effective.

To sum up, the advantages of the presented PL-CoCoSo method can be highlighted as follows:

1. The subordinate performance values obtained from each aggregation strategy are fully considered, which conforms to the idea of compromise;
2. The subordinate performance values and subordinate ranks are considered at the same time to make the final results reliable; and
3. The proposed method has a wide scope of applications. It can be used to solve the decision-making problems in which the criteria are a cost type, benefit type, or both cost and benefit types. The method is easy to calculate and understand in solving the decision-making problems with many alternatives and criteria.

## 6. Conclusions

Cancer has seriously endangered human health and has a high mortality rate. Because medicines are the main means to prevent and treat cancer, it is vital to ensure the quality of medicines during transportation and storage, which makes it of great significance for most drug manufacturers to evaluate and select a drug cold chain logistics supplier from the perspective of risk aversion. To solve this problem, an integrated MCDM model was proposed in this paper. In this MCDM model, we extended the SWARA method and improved the CoCoSo method to a probabilistic linguistic environment. Then, the proposed integrated MCDM model was used to solve the decision-making problem of selecting drug cold chain logistics suppliers. By the case study, we highlighted the simplicity of the PL-SWARA method in deriving the weights of risk evaluation criteria with respect to cold chain logistics suppliers, and the reliability of the PL-CoCoSo method in determining the ranking of cold chain logistics suppliers.

In this paper, the comparison between the proposed method and other MCDM methods was still inadequate. For future research, we will consider applying the proposed method to solve decision-making problems in other fields to enhance the applicability of this method, and compare this method with other methods to further analyze the advantages and disadvantages of this method. In addition, we will explore other MCDM models to solve the decision-making problem in the field of drug cold chain logistics.

## Figures and Tables

**Table 1:** The application of the SWARA method in different fields.

Application Field	Authors	Main Contributions
**Management**	Zarbakhshnia et al. [27]	Select the third-party reverse logistics suppliers to realize supply chain management
	Karabasevic et al. [28]	Make a personal selection with the SWARA method
**Engineering**	Hashemkhani Zolfani et al. [29]	Determining the weights of criteria for shopping mall selection
	Vafaeipour et al. [30]	Solve the problem of site selection for sustainable solar power plant construction
	Ruzgys et al. [31]	Choose a suitable housing modernization program to reduce the energy consumption of old houses and achieve sustainable development
**Manufacturing**	Shukla et al. [32]	Evaluate the enterprise resource planning (ERP) to select an ERP system suitable for the enterprise environment
	Aghdaie et al. [33]	Choose the machine tools used in manufacturing to improve the market competitiveness of enterprises
	Stanujkic et al. [34]	Choose packaging design scheme in view of customers’ demand
**Others**	Keršulienė et al. [17]	Choose the method to settle legal disputes
	Hashemkhani Zolfani and Bahrami [35]	Decision making on priority development of Iranian high-tech industry

**Table 2:** Risk evaluation criteria for drug cold chain logistics suppliers.

	Criteria	Main Causes of Risks
**Storage process**	$c_{1}$: Storage maintenance planning risk	Improper storage and maintenance planning
	$c_{2}$: Temperature and humidity monitoring risk	Backward monitoring technology; Over-long monitoring interval design; Inadequate work of responsible personnel
	$c_{3}$: Validity period management risk	Technological backwardness; The query interval is too long
**Transportation process**	$c_{4}$: Delivery planning risk	Unreasonable planning
	$c_{5}$: Equipment failure risk	Backward equipment; Failure to inspect and repair the equipment in time
	$c_{6}$: Traffic environmental risk	Traffic jams; Accidents
	$c_{7}$: Temperature control risk	Lack of staff’s sense of responsibility; Backward equipment
	$c_{8}$: Customer acceptance risk	Customer acceptance personnel operation is not standardized

Source: The research of Chatterjee and Pandey [2] on the risk management of the drug cold chain logistics process. Note: Due to the evaluation on risks, the criteria here are all cost-type criteria.

**Table 3:** The result derived by the PL-SWARA method.

	$D_{1}$	$D_{2}$	$D_{3}$	$D_{4}$	$z_{j}^{*}$	$w_{j}^{\prime }$	$AC_{j}$	$w_{j}$
$c_{2}$	--	--	--	--	1	1	1	0.351
$c_{7}$	0.925	0.75	1	0.875	0.525	0.656	0.87	0.2
$c_{1}$	0.5	0.4	0.5	0.25	0.244	0.527	0.855	0.158
$c_{4}$	1	0.95	0.938	1	0.575	0.335	0.956	0.112
$c_{5}$	0.6	0.5	0.5	0.8	0.355	0.247	0.813	0.07
$c_{3}$	0.75	0.813	0.55	0.5	0.386	0.178	0.748	0.047
$c_{6}$	0.5	0.25	0.5	0.125	0.203	0.148	0.608	0.032
$c_{8}$	0.35	0.25	0.125	0.175	0.133	0.131	0.662	0.03

**Table 4:** The results obtained by the PL-CoCoSo method.

	$AW_{i}$	$GW_{i}$	$T_{i}^{1*}$	Ranks	$T_{i}^{2*}$	Ranks	$T_{i}^{3*}$	Ranks	$T_{i}^{*}$	Ranks
$A_{1}$	0.706	6.729	0.186	3	3.69	1	0.965	2	2.556	2
$A_{2}$	0.701	6.762	0.187	1	3.681	2	0.966	1	2.688	1
$A_{3}$	0.587	5.646	0.156	5	3.079	3	0.807	4	1.926	4
$A_{4}$	0.325	6.117	0.161	4	2.352	5	0.749	5	1.68	5
$A_{5}$	0.316	4.622	0.124	6	2	6	0.595	6	1.288	6
$A_{6}$	0.379	7.076	0.187	1	2.73	4	0.868	3	2.221	3

**Table 5:** The results computed by the three aggregation strategies in the HFL-CoCoSo method.

	$AW_{i}^{\prime }$	$GW_{i}^{\prime }$	$T_{i}^{1^{\prime }}$	Ranks	$T_{i}^{2^{\prime }}$	Ranks	$T_{i}^{3^{\prime }}$	Ranks
$A_{1}$	0.757	6.805	0.19	2	4.021	1	0.932	2
$A_{2}$	0.711	7.519	0.207	1	4.014	2	0.983	1
$A_{3}$	0.596	5.679	0.158	4	3.233	3	0.764	4
$A_{4}$	0.292	4.937	0.132	5	2.037	5	0.578	5
$A_{5}$	0.293	4.763	0.127	6	2.003	6	0.563	6
$A_{6}$	0.368	7.038	0.186	3	2.738	4	0.807	3

**Table 6:** The results determined by the aggregation approach in the HFL-CoCoSo method.

	$PS_{1}(A_{i})$	$R_{1}(A_{i})$	$PS_{2}(A_{i})$	$R_{2}(A_{i})$	$PS_{3}(A_{i})$	$R_{3}(A_{i})$	$R(A_{i})$	Ranks
$A_{1}$	2.385	2	2.222	1	1.803	2	5	2
$A_{2}$	2.222	1	2.385	2	1	1	4	1
$A_{3}$	3.683	4	2.883	3	3.5	4	11	4
$A_{4}$	4.507	5	4.507	5	4.359	5	15	5
$A_{5}$	5.344	6	5.344	6	5.22	6	18	6
$A_{6}$	2.883	3	3.683	4	2.646	3	10	3

## Appendix A

The steps of the SWARA method can be described as follows:

**Step 1.** Sort the set of criteria in descending order according to their importance, and obtain the ranking result as ($c_{1},c_{2},\cdots ,c_{j},\cdots ,c_{n}$).

**Step 2.** The relative importance of criterion $c_{j}\;(j>1)$ with regard to its previous criterion, $c_{j-1}$, is expressed by the expert as $z_{j}$.

**Step 3.** Calculate the coefficient, $p_{j}$, by:

(A1) $$p_{j}=\{\begin{array}{ll} 1 & ,\;\mathrm{for}\;j=1\\ z_{j}+1 & ,\;\mathrm{for}\;j>1 \end{array}.$$

**Step 4.** Compute the weight ${\hat{w}}_{j}$ by:

(A2) $${\hat{w}}_{j}=\{\begin{array}{ll} 1 & ,\;\mathrm{for}\;j=1\\ p_{j-1}/p_{j} & ,\;\mathrm{for}\;j>1 \end{array}.$$

**Step 5.** To make the weight sum of all criteria equal to 1, the final criterion weight, $w_{j}$, is obtained by:

(A3) $$w_{j}={\hat{w}}_{j}/\sum_{j=1}^{n}{\hat{w}}_{j}.$$

## Appendix B

The steps of the CoCoSo method are summarized as follows:

**Step 1.** Normalize the initial decision matrix by:

(A4) $$N_{ij}=\{\begin{array}{c} (x_{ij}-\underset{i}{\min}x_{ij})/(\underset{i}{\max}x_{ij}-\underset{i}{\min}x_{ij}),\mathrm{for}\;\mathrm{benifit}\;\mathrm{criteria}\\ (\underset{i}{\max}x_{ij}-x_{ij})/(\underset{i}{\max}x_{ij}-\underset{i}{\min}x_{ij}),\mathrm{for}\;\cos t\;\mathrm{criteria} \end{array},$$

where $a_{i}\;(i=1,2,\cdots ,m)$ are alternatives and $c_{j}$(j=1,2,⋯,n) are criteria.

**Step 2.** Calculate the overall performance values of each alternative $PV_{i}^{1}$, $PV_{i}^{2}$ by Equations (A5) and (A6), respectively:

(A5) $$PV_{i}^{1}=\sum_{j=1}^{n}(w_{j}N_{ij}),$$

(A6) $$PV_{i}^{2}=\sum_{j=1}^{n}{\left(N_{ij}\right)}^{w_{j}},$$

where $w_{j}(j=1,2,\cdots ,n)$ are the weights of criteria, the sum of which equals 1.

**Step 3.** Combine the two overall performance values of alternatives by three aggregation strategies as follows:

(A7) $$T_{i}^{1}=\frac{PV_{i}^{1}+PV_{i}^{2}}{\sum_{i=1}^{m}\left(PV_{i}^{1}+PV_{i}^{2}\right)},$$

(A8) $$T_{i}^{2}=\frac{PV_{i}^{1}}{\underset{i}{\min}PV_{i}^{1}}+\frac{PV_{i}^{2}}{\underset{i}{\min}PV_{i}^{2}},$$

(A9) $$T_{i}^{3}=\frac{\delta (PV_{i}^{1})+(1-\delta )(PV_{i}^{2})}{\delta \underset{i}{\max}PV_{i}^{1}+(1-\delta )\underset{i}{\max}PV_{i}^{2}},$$

where the parameter δ in Equation (10) plays a role in balancing two kinds of overall performance values.

$T_{i}^{1}$, $T_{i}^{2}$, and $T_{i}^{3}$ respectively represent the three subordinate compromise performance values of alternative i.

**Step 4.** Calculate the compromise performance values of alternatives $T_{i}$ by the final integration function shown as Equation (11), and obtain the final ranking of the alternative:

(A10) $$T_{i}=\frac{1}{3}(T_{i}^{1}+T_{i}^{2}+T_{i}^{3})+{\left(T_{i}^{1}T_{i}^{2}T_{i}^{3}\right)}^{\frac{1}{3}}.$$

## Appendix C

**Table A1:** The linguistic evaluation information of the alternatives given by $D_{1}$.

	$A_{1}$	$A_{2}$	$A_{3}$	$A_{4}$	$A_{5}$	$A_{6}$
$c_{1}$	Between *medium* and *slightly high*	Between *slightly high* and *high*	*Very high*	Between *slightly low* and *slightly high*	*High*	*Slightly high*
$c_{2}$	Between *slightly high* and *very high*	*Slightly high*	*High*	At least *very high*	*Very high*	Between *high* and *very high*
$c_{3}$	*Slightly low*	Between *very low* and *slightly low*	*Slightly low*	Between *slightly low* and *medium*	Between *medium* and *slightly high*	Between *low* and *slightly low*
$c_{4}$	Between *low* and *medium*	Between *slightly low* and *medium*	Between *low* and *slightly low*	*Medium*	Between *low* and *medium*	*Slightly low*
$c_{5}$	Between *medium* and sh	Between *low* and *slightly low*	Between *very low* and *slightly low*	Between *very low* and *low*	*Slightly high*	Between *slightly low* and *medium*
$c_{6}$	Between *low* and *slightly low*	Between *very low* and *low*	*Medium*	Between *slightly low* and *medium*	Between *medium* and *slightly high*	Between *very low* and *slightly low*
$c_{7}$	Between *slightly low* and *medium*	*Slightly high*	Between *slightly high* and *high*	Between *high* and vh	Between *medium* and *slightly high*	Between *slightly high* and *high*
$c_{8}$	At most *very low*	*Low*	Between *low* and *slightly low*	At most *low*	*Slightly low*	Between *very low* and *slightly low*

**Table A2:** The linguistic evaluation information of the alternatives given by $D_{2}$.

	$A_{1}$	$A_{2}$	$A_{3}$	$A_{4}$	$A_{5}$	$A_{6}$
$c_{1}$	*Slightly high*	Between *medium* and *high*	*High*	Between *slightly low* and *medium*	*Slightly high*	Between *slightly high* and *high*
$c_{2}$	Between *high* and *very high*	*Slightly high*	Between *slightly high* and *high*	*High*	At least *high*	Between *high* and *very high*
$c_{3}$	*Low*	Between *low* and *slightly low*	Between *medium* and *slightly high*	Between *slightly low* and *medium*	*Medium*	*Slightly low*
$c_{4}$	Between *slightly low* and *medium*	*Slightly low*	Between *very low* and *low*	*Medium*	Between *slightly low* and *medium*	*Slightly low*
$c_{5}$	*Medium*	Between *slightly low* and *medium*	*Slightly low*	Between *low* and *slightly low*	Between *slightly low* and *medium*	*Medium*
$c_{6}$	Between *low* and *slightly low*	Between *very low* and *slightly low*	Between *slightly low* and *medium*	Between *slightly low* and *slightly high*	*High*	Between *very low* and *low*
$c_{7}$	Between *medium* and *slightly high*	Between *slightly high* and *high*	Between *slightly high* and *very high*	Between *high* and *very high*	Between *slightly low* and *slightly high*	*Slightly high*
$c_{8}$	At most *very low*	*Slightly low*	Between *low* and *slightly low*	At most *very low*	Between *very low* and *low*	Between *very low* and *slightly low*

**Table A3:** The linguistic evaluation information of the alternatives given by $D_{3}$.

	$A_{1}$	$A_{2}$	$A_{3}$	$A_{4}$	$A_{5}$	$A_{6}$
$c_{1}$	Between *slightly low* and *medium*	*medium*	At least *high*	Between *medium* and *slightly high*	*Slightly high*	Between *medium* and *high*
$c_{2}$	Between *slightly high* and *very high*	*Slightly high*	Between *medium* and *slightly high*	*Very high*	At least *high*	*High*
$c_{3}$	*Slightly low*	Between *very low* and *slightly low*	Between *slightly low* and *medium*	Between *slightly low* and *slightly high*	Between *medium* and *slightly high*	Between *low* and *slightly low*
$c_{4}$	Between *low* and *slightly low*	Between *medium* and *slightly high*	*Slightly low*	Between *slightly low* and *medium*	Between *low* and *medium*	*Medium*
$c_{5}$	Between *medium* and *slightly high*	Between *slightly low* and *medium*	Between *very low* and *slightly low*	*Low*	*Slightly low*	Between *slightly low* and *medium*
$c_{6}$	*Low*	Between *low* and *slightly low*	*Slightly low*	Between *medium* and *slightly high*	Between *slightly high* and *high*	Very low
$c_{7}$	Between *slightly low* and *slightly high*	Between *medium* and *high*	Between *high* and vh	*High*	*Slightly high*	Between *slightly high* and vh
$c_{8}$	At most *very low*	*Very low*	Between *slightly low* and *medium*	At most *low*	Between *low* and *slightly low*	Between *very low* and *low*

**Table A4:** The linguistic evaluation information of the alternatives given by $D_{4}$.

	$A_{1}$	$A_{2}$	$A_{3}$	$A_{4}$	$A_{5}$	$A_{6}$
$c_{1}$	Between *medium* and *slightly high*	Between *slightly high* and *high*	Between *high* and *very high*	*Slightly high*	*High*	Between *slightly high* and *high*
$c_{2}$	Between *slightly high* and *very high*	*Slightly high*	*High*	At least *high*	At least *very high*	*very high*
$c_{3}$	Slightly *low*	Between *very low* and *slightly low*	*Slightly low*	Between *slightly low* and *medium*	Between *medium* and *slightly high*	*Low*
$c_{4}$	Between *low* and *medium*	Between *slightly low* and *medium*	Between *low* and *slightly low*	*Medium*	Between *low* and *medium*	Between *low* and *medium*
$c_{5}$	Between medium and slightly high	Between *low* and *slightly low*	Between *very low* and *slightly low*	Between *very low* and *low*	*Slightly high*	Between *slightly low* and *slightly high*
$c_{6}$	Between *low* and *slightly low*	Between *very low* and *low*	*Medium*	*Slightly low*	*High*	Between *low* and *slightly low*
$c_{7}$	Between *slightly low* and *medium*	*Slightly high*	Between *slightly high* and vh	At least *high*	Between *medium* and *slightly high*	Between *high* and *very high*
$c_{8}$	At most *very low*	*Very low*	Between *low* and *slightly low*	At most *very low*	Between *low* and *slightly low*	Between *very low* and *low*

**Table A5:** The PLEs about the preference information for criteria provided by the experts.

	$D_{1}$	$D_{2}$	$D_{3}$	$D_{4}$
$c_{2}$	--	--	--	--
$c_{7}$	$\left\{s_{3}(0.3),\;s_{4}(0.7)\right\}$	$\left\{s_{3}(0.7)\right\}$	$\left\{s_{4}(1)\right\}$	$\left\{s_{3}(0.5),\;s_{4}(0.5)\right\}$
$c_{1}$	$\left\{s_{2}(0.8)\right\}$	$\left\{s_{1}(0.4),\;s_{2}(0.6)\right\}$	$\left\{s_{2}(0.9)\right\}$	$\left\{s_{1}(0.6)\right\}$
$c_{4}$	$\left\{s_{4}(0.6)\right\}$	$\left\{s_{3}(0.2),\;s_{4}(0.8)\right\}$	$\left\{s_{3}(0.2),\;s_{4}(0.6)\right\}$	$\left\{s_{4}(0.7)\right\}$
$c_{5}$	$\left\{s_{2}(0.6),\;s_{3}(0.4)\right\}$	$\left\{s_{2}(1)\right\}$	$\left\{s_{2}(0.8)\right\}$	$\left\{s_{4}(0.2),\;s_{3}(0.8)\right\}$
$c_{3}$	$\left\{s_{3}(0.8)\right\}$	$\left\{s_{3}(0.6),\;s_{4}(0.2)\right\}$	$\left\{s_{2}(0.8),\;s_{3}(0.2)\right\}$	$\left\{s_{2}(0.6)\right\}$
$c_{6}$	$\left\{s_{2}(0.7)\right\}$	$\left\{s_{1}(1)\right\}$	$\left\{s_{2}(1)\right\}$	$\left\{s_{0}(0.3),\;s_{1}(0.3)\right\}$
$c_{8}$	$\left\{s_{1}(0.6),\;s_{2}(0.4)\right\}$	$\left\{s_{1}(0.8)\right\}$	$\left\{s_{0}(0.5),\;s_{1}(0.5)\right\}$	$\left\{s_{0}(0.3),\;s_{1}(0.7)\right\}$
